# A universal framework for IMRT dose prediction

**DOI:** 10.1002/mp.70384

**Published:** 2026-03-15

**Authors:** Qingying Wang, Mingli Chen, Yinheng Zhu, Mahdieh Kazemimoghadam, Kangning Zhang, Zi Yang, Hao Jiang, Xuejun Gu, Weiguo Lu

**Affiliations:** ^1^ Department of Radiation Oncology University of Texas Southwestern Medical Center Dallas Texas USA; ^2^ Department of Radiation Oncology Stanford University Stanford California USA

**Keywords:** deep learning, dose prediction, radiation therapy

## Abstract

**Background:**

Dose prediction has great potential in improving plan quality and efficiency by estimating optimal dose distribution. However, most existing deep learning (DL) based dose prediction models for intensity‐modulated radiation therapy (IMRT) have been primarily developed under simplified conditions, such as fixed beam configuration and/or disease site. These constraints limit the generalizability and clinical usability of such models across the diverse scenarios encountered in real‐world practice.

**Purpose:**

We proposed a DL‐based universal dose prediction model, named UniDose, designed to accommodate a wide range of disease sites and support diverse clinical scenarios, especially for IMRT treatment plans with arbitrary beam configurations.

**Methods:**

UniDose is built on a customized nnU‐Net framework, adapted into an image‐to‐image mapping network tailored for 3D dose prediction and trained using the Huber loss. The network takes three generalized input channels: a normalized prescription dose map that encodes planning goals for the target, a weighted avoidance mask that consolidates multiple organs at risk (OARs) and body structures into a single channel with clinical relevance–based voxel weights, and a beam trace image that captures beam configuration using a non‐modulated, cumulative dose approximation generated via a ray‐tracing based algorithm. The model was trained, validated and tested on a heterogeneous dataset of 871 patients encompassing 25 disease sites and a wide spectrum of prescription doses and beam configurations. To assess the deliverability of the predicted dose, we incorporated a reference‐based in‐house optimization engine into the UniDose framework to generate feasible plans constrained by machine limitations. Model performance was evaluated by comparing predicted doses, optimized doses, and clinical plans using gamma passing rate (GPR) with a 3%/2 mm criteria and 10% lower dose threshold and dose‐volume histogram (DVH) metrics.

**Results:**

The UniDose predictions achieved an average GPR of 92.36% compared to the optimized doses and demonstrated strong DVH consistency. The average GPR between predicted and clinical doses was 86.13%. DVH comparisons showed that the predictions and the optimized dose achieved improved OAR sparing while maintaining comparable target coverage relative to clinical dose, particularly in prostate, liver, and brain cases. Case studies across six disease sites with variable beam configurations further confirmed that the predicted and optimized doses exhibited similar dose deposition patterns along beam paths, suggesting that the predicted dose is physically feasible and approachable following dose optimization. Additionally, adjusting voxel weights in the avoidance input channel enabled flexible trade‐offs between OAR sparing and target coverage, supporting patient‐specific treatment planning.

**Conclusions:**

UniDose demonstrates strong potential as a universal DL‐based dose prediction framework capable of generalizing across diverse disease sites and beam configurations. By combining a generalized input design, robust network customization, and integration with a reference‐guided optimization engine, UniDose generates physically feasible dose predictions and allows efficient user interaction through adjustable input conditions.

## 1 INTRODUCTION

Intensity modulated radiation therapy (IMRT) is a modern external radiotherapy technique that delivers highly conformal doses by modulating beam intensities from multiple directions to maximize tumor coverage while sparing nearby organs at risk (OARs).^1^, ^2^ The planning of IMRT is a complex process that requires collaboration among multi‐disciplinary teams to determine optimal beam configurations and intensity settings for each patient. Traditionally, treatment planners manually adjust multiple weighting parameters through a trial‐and‐error approach to balance conflicting clinical objectives, making the process both time‐consuming and labor‐intensive.^3^, ^4^ Furthermore, even under the same planning mode, variations in tumor location and patient anatomy lead to patient‐specific strategies with varying levels of difficulty. Thus, the quality of the final plan often depends heavily on the expertise of the planner. These challenges have motivated the development of numerous automated planning techniques to improve workflow efficiency and ensure consistency in plan quality.^5^, ^6^, ^7^, ^8^, ^9^, ^10^, ^11^, ^12^

Current auto‐planning methods generally follow three main directions: multi‐criteria optimization (MCO),^13^ automated iterative planning (AIP),^8^, ^14^ and dose prediction (DP) models which include both traditional knowledge‐based planning (KBP) and deep learning (DL)‐based approaches.^5^, ^7^, ^9^, ^10^, ^11^, ^12^ For DP, a system trained on a set of clinically approved plans is used to infer dose‐related information for a new patient based on input features such as anatomy and beam configuration. Compared to MCO and AIP, DP models are appealing for their ability to efficiently predict dose‐volume histograms (DVHs),^15^ dose‐volume objectives,^5^ or dose distributions^9^ as planning guidance within seconds per case, as they bypass the need to solve optimization problems during inference. The time cost of DP models occurs primarily during data preparation and model training due to their inherently data‐driven nature. The DL‐based DP method offers further advantages over KBP, as it does not rely on handcrafted features or predefined statistical models, and can better generalize beyond limited clinical scenarios in the collected dataset.

While DL‐based DP has made significant progress, improving clinical applicability and usability remains a critical challenge. Most existing studies focus on enhancing prediction performance through designing new neural network architectures,^16^ introducing novel loss functions,^17^ or integrating additional prior knowledge into the model.^18^, ^19^ Model performance is typically quantified by comparing predictions to a designated ground truth, such as a clinically approved plan or one generated under standardized quality control. However, this evaluation primarily reflects how well the model replicates the provided reference data and may not reliably assess its clinical utility. A more comprehensive evaluation is needed. First, the predicted dose distribution may be unrealistic, as it does not account for delivery constraints imposed by the treatment machine. Second, treatment planning is inherently a multi‐objective problem with no single optimal solution, but rather a set of Pareto optimal trade‐offs. Therefore, evaluating prediction performance solely based on similarity to a single reference plan can be both limited and potentially misleading. To facilitate real‐world translation, it is essential to evaluate the quality of the predicted dose in terms of both physical feasibility, which ensures deliverability within system constraints, and dosimetric quality, which reflects clinically meaningful and acceptable trade‐offs.

Real‐world clinical scenarios are typically highly heterogeneous, especially in the context of IMRT treatment planning, encompassing diverse disease sites, prescription doses, and beam configurations. Therefore, a higher‐level requirement for DL‐based DP model is to improve the generalizability, enabling it to adapt to a broader range of clinical scenarios while providing accurate, patient‐specific predictions. However, previous DL‐based models have primarily been developed and evaluated under simplified conditions, such as consistent beam configurations and fixed disease sites. For example, most existing approaches rely on common input channels, including computed tomography (CT) images, organ‐at‐risk (OAR) masks, and target volume masks, without incorporating any information about beam delivery patterns.^10^, ^16^, ^20^, ^21^ Consequently, these models rely on a strong assumption that treatment plans follow consistent beam configurations, and therefore struggle to generalize to cases with unseen or arbitrary beam arrangements. Although recent studies have attempted to incorporate multi‐beam geometry information to develop more generalizable models,^11^, ^12^, ^22^, ^23^, ^24^ they have mostly been limited to specific disease sites, such as rectum,^22^ lung,^11^, ^12^, ^24^ and brain,^23^ where the spatial relationships between the target and OARs remain relatively consistent. Moreover, treating OARs as individual inputs^9^, ^11^, ^12^, ^16^ limits model generalizability and practicality, as clinical datasets are often inconsistent that only partially labeled. As a result, the challenge of generalizing across both diverse multi‐beam geometries and heterogeneous anatomical sites remains largely unaddressed.

In this work, we propose a universal DL‐based DP model named UniDose for diverse disease sites with arbitrary beam configurations. UniDose is built upon a customized nnU‐Net framework,^25^ which is originally designed for segmentation tasks but offers strong adaptability to diverse datasets. To leverage this advantage, we customized nnU‐Net^25^ into an image‐to‐image mapping network tailored for continuous dose distribution prediction. The input design of UniDose is also generalized, incorporating patient anatomy and beam setup information to guide the inference of 3D dose distributions. To assess the physical feasibility of the predicted dose, we further perform dose optimization using an in‐house optimization engine driven by the UniDose prediction. We evaluate UniDose on a highly heterogeneous dataset spanning a broad range of disease sites and beam configurations to demonstrate its generalizability and clinical applicability.

## 2 MATERIALS AND METHODS

### 2.1 Patient database

**FIGURE 1:**
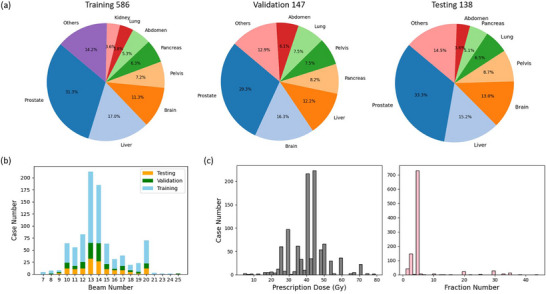
Overview of the patient dataset with respect to disease sites, beam configurations, prescription doses, and treatment fractions. (a) Distribution of disease sites across the training (*n* = 586), validation (*n* = 147), and testing (*n* = 138) cohorts. (b) Number of patients per beam configuration, ranging from 7 to 25 beams, in each dataset split. (c) Distribution of prescription doses (4–79.2 Gy) and treatment fractions (1–44 fractions).

In this retrospective study, we conducted experiments on a large and heterogeneous dataset comprising 871 patients treated with IMRT, collected from University of Texas Southwestern Medical Center radiotherapy database with IRB approval. Details of the patient are illustrated in Figure 1. The dataset spans 25 disease sites, with prostate, liver, and brain cases accounting for over 50% of the cohort. Disease sites representing fewer than 3% of the total, such as the eye, bile duct, and leg, are grouped under “Others,” as shown in Figure 1a. The number of beams per plan ranges from 7 to 25 with arbitrary orientations, most commonly configurations involving 13 or 14 beams, as shown in Figure 1b. All treatment plans were manually designed with various prescriptions and delivered with different fractionation protocols, as shown in Figure 1c. Additional details on the dataset composition can be found in Tables S1–S3. All clinically approved dose distributions were designed, optimized, and calculated from the Monaco treatment planning system (Elekta AB, Stockholm, Sweden). Targets and OARs were delineated by radiation oncologists/medical physicists. The clinically approved dose distributions and contour masks for all patients were resampled to a 2 mm isotropic resolution.

### 2.2 UniDose for dose prediction

**FIGURE 2:**
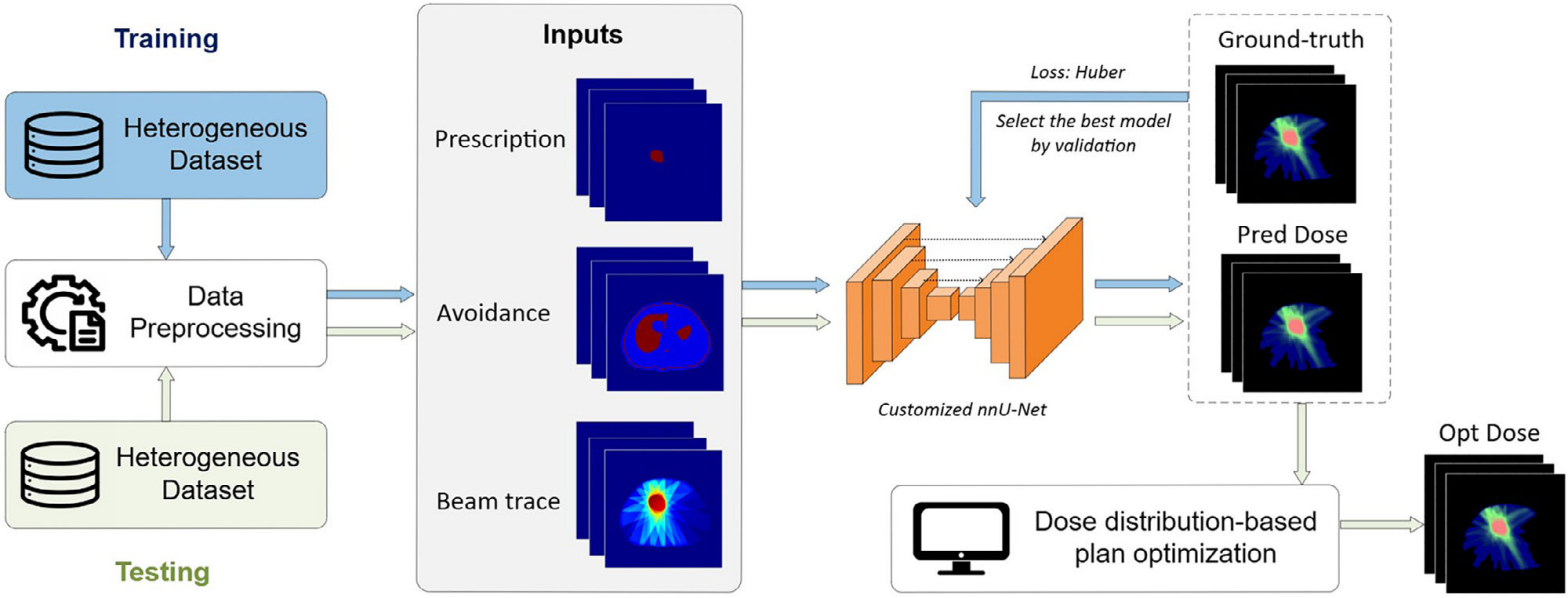
Overview of the UniDose workflow. A customized nnU‐Net predicts 3D dose distributions from three input channels using a Huber loss. The predicted dose (Pred Dose) guides a plan optimization process to generate an optimized dose (Opt Dose) distribution.

The UniDose framework is built upon a customized nnU‐Net architecture, trained to learn the mapping between voxel‐wise anatomical geometry, multi‐beam geometry, and corresponding dose distribution. As shown in Figure 1, the training, validation, and testing datasets used in UniDose are highly heterogeneous, encompassing a wide range of disease sites and multi‐beam geometry. To ensure robust learning across these diverse cases, the data preprocessing pipeline standardizes raw patient data into three generalized input channels for model training. To mitigate overfitting, model selection is based on the best‐performing checkpoint on the validation set. During inference, an optimized dose distribution is generated based on the predicted dose distribution and subsequently compared against both the clinically planned dose and the predicted dose. The overall workflow of UniDose is depicted in Figure 2.

#### 2.2.1 Data pre‐processing

**FIGURE 3:**
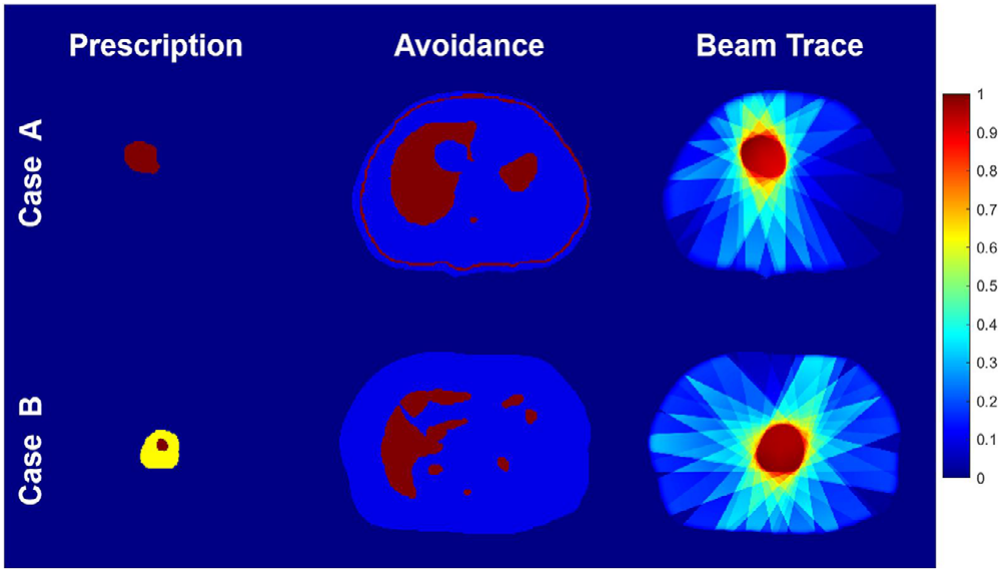
Illustration of the three input channels: prescription, avoidance, and beam trace for Case A (single‐prescription dose) and Case B (simultaneous integrated boost, SIB).

The three input channels for UniDose were deliberately designed and preprocessed to be generalizable, enabling robust representation of target prescription, OAR avoidance, and beam directions configuration across diverse and heterogeneous clinical datasets, as illustrated in Figure 3.

The first channel is the normalized target prescription dose image, referred to as prescription, which serves as a surrogate for the clinical planning goal by representing an ideal dose distribution. It encodes the dose values within the planning target volume (PTV), normalized to the maximum prescribed dose to ensure consistency and comparability across different prescription levels. For instance, in Figure 3, Case A with a single‐prescription dose, the prescription channel is represented as a binary mask of the PTV. In contrast, in Case B with simultaneous integrated boost (SIB), the prescription is normalized to the maximum prescribed dose, producing two discrete value levels within the PTV that correspond to the different dose prescriptions.

The second channel is the weighted avoidance mask image, referred to as avoidance, which highlights regions critical for dose sparing by consolidating multiple OARs and body contours into a single map. Unlike traditional dose prediction models that use separate channels for each OAR,^9^, ^11^, ^12^, ^16^ this unified representation enhances practicality, particularly for clinical datasets that numbers and types of OAR labeling are not unified. Voxel weights in the avoidance map are assigned based on clinical relevance: OAR voxels are set to 1, body voxels to 0.1, and all other voxels to 0.

The third input channel encodes the multi‐beam geometry, referred to as the beam trace map. To include this information without adding model complexity, the geometry is represented as a normalized dose distribution map, following a processing approach similar to that described in a previous study.^11^ Specifically, we generate this map using a ray tracing algorithm with beam apertures conformal to the PTV in each beam's eye view and a unit‐fluence map. Dose contributions are then computed using the fluence‐convolution broad‐beam (FCBB)^26^ performing a 2D convolution on each beam aperture with a 5 mm isotropic margin and ray tracing^27^ with percentage depth dose derived from machine commissioning data. The algorithm is capable of generating the dose per beam in a sub‐second. The resulting per‐beam doses are then summed and normalized by the total number of beams, yielding a beam trace map that represents relative beam‐path dose contributions independent of beam count, with the maximum value typically close to 1, as illustrated in Figure 3. Compared with a beam angle map that only encodes beam directions, the beam trace map provides a more physically meaningful representation by approximating the fundamental 2D to 3D dose calculation process. Using a unit fluence map, the beam trace encodes cumulative dose characteristics through ray tracing and energy deposition along the beam path, capturing key beam‐dependent features such as direction, energy dependent depth‐dose behavior, and attenuation related to radiological path length. This physics‐inspired representation offers a coarse but informative approximation of expected dose deposition in three dimensions, reducing the burden on the network to infer this relationship purely from beam geometric information and enabling effective learning across arbitrary beam configurations.

#### 2.2.2 Dose prediction network and experimental setting

In our previous study, we demonstrated the potential of nnU‐Net^25^ to perform dose prediction as an off‐the‐shelf tool, without requiring changes to its architecture.^7^ While this approach achieved promising results, it was based on a strong assumption that all cases shared consistent beam configurations and required post‐processing to convert the discrete segmentation‐style dose prediction into a continuous dose distribution, limiting its generalizability and clinical applicability in more complex scenarios.

Therefore, in this work, we customize the nnU‐Net framework to support voxel‐wise regression and the original classification loss is replaced with the Huber loss,^28^ as defined in Equation (1), where $\hat{d}$ and d denote the prediction and ground‐truth, respectively, and the threshold was set to δ=1. The Huber loss provides a balance between sensitivity to outliers and training stability by combining the properties of mean squared error and mean absolute error. All other aspects of nnU‐Net, including the network architecture, dynamic adaptation to the dataset, preprocessing, and data augmentation strategies, are unchanged from the default values of the original implementation.^25^ This allows us to maintain the robustness and reproducibility of nnU‐Net while extending its capability to produce accurate, patient‐specific dose distributions across heterogeneous clinical settings.

(1) $$L_{\delta }\;\left(\hat{d}-d\right)=\left\{\begin{array}{c} \frac{1}{2}{\left(\hat{d}-d\right)}^{2},\;if\;\left|\hat{d}-d\right|\leq \delta ,\\ \delta |\hat{d}-d|-\frac{1}{2}\delta^{2},\;otherwise. \end{array}\;\right.\;$$

The Windows version of nnU‐Net was downloaded from the GitHub repository (https://github.com/marcuswirtz‐snkeos/nnUNet) and compiled into standalone executables using PyInstaller (https://pyinstaller.org/en/stable/). Compilation was performed on a development machine configured with a Python environment that meets the requirements of the model. The resulting executables were then transferred to a test workstation equipped with an NVIDIA GeForce RTX 2080 Ti GPU and an Intel Core i7‐9700K CPU, but without a Python environment. All training and inference processes were conducted using these precompiled executables, allowing the customized nnU‐Net to function as an off‐the‐shelf application. The training process for UniDose was terminated at 1000 epochs with a converged loss score, requiring approximately 19 h in total. The average inference time was around 1.5 s per case.

### 2.3 Performance evaluation

While dose prediction provides a fast, data‐driven estimate of the expected dose distribution, such predictions are not guaranteed to be physically approachable. Therefore, evaluating the physical feasibility of the predicted dose is critical before clinical translation. One way to assess this is by comparing the predicted dose with an optimized dose that uses the prediction as a reference. Unlike pure prediction, the optimized dose is physics‐driven, obtained by explicitly solving the fluence map optimization (FMO) problem under the dose–fluence relationship. This process ensures that the optimized dose distribution is achievable through non‐negative beamlet intensities and satisfies the optimization objective function. Thus, if the predicted and optimized doses are closely aligned, it demonstrates that the prediction model not only captures clinical trade‐offs but also approximates a physically feasible treatment plan. In this work, we integrated an in‐house threshold‐driven optimization for reference‐based auto‐planning (TORA) framework^29^ into the UniDose workflow to generate the optimized dose, as illustrated in Figure 2. TORA uses the predicted dose as a reference and is coupled with the non‐voxel‐based broad‐beam (NVBB) framework,^30^ which supports large‐scale IMRT optimization without requiring pre‐calculated beamlets. The average optimization time was 37.7 ± 8.1 s per case, demonstrating that the optimization stage remains computationally efficient.

The gamma passing rate (GPR), using 3%/2 mm criteria with a 10% low‐dose threshold (LDT), was used to assess agreement between the predicted dose, optimized dose, and clinical dose. For DVH‐based evaluation, the following metrics were analyzed: $D_{5\%}$ and $D_{95\%}$ for target coverage, and $D_{5\%}$ and $D_{50\%}$ for OARs dose sparing, where $D_{x\%}$ represents the dose that x% of the volume of a ROI is at least receiving. To quantify deviations, the mean absolute percentage error (MAPE) was calculated for each DVH metric by comparing the predicted dose with the optimized or clinical dose, as defined in Equation (2), with $D_{P}$ as the prescribed dose and n is the number of cases in the study.

(2) $$\mathrm{MAPE}\left(\%\right)\;=\;\frac{100\%}{n}\sum_{i=1}^{n}\left|\frac{D_{x\%}^{i}\left(\mathrm{Pred}\right)\;-\;D_{x\%}^{i}\left(\mathrm{Opt}\;\mathrm{or}\;\mathrm{Clin}\mathrm{ical}\right)}{D_{p}}\right|$$

## 3 RESULTS

**FIGURE 4:**
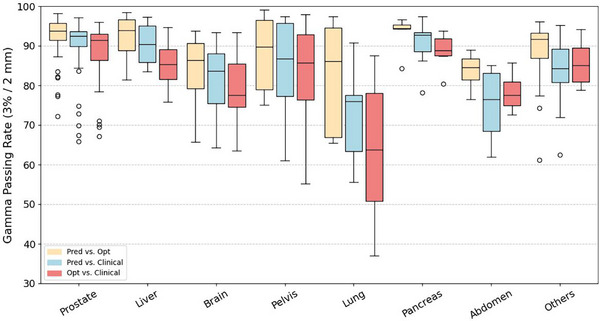
The plot of gamma passing rate (GPR) with 3%/2 mm criteria and 10% low dose threshold (LDT) assessing the agreement of predicted and optimized dose (Pred vs. Opt), predicted and clinical dose (Pred vs. Clinical), and optimized and clinical dose (Opt vs. Clinical) on various disease sites.

In the statistical evaluation, the 3%/2 mm GPR with a 10% LDT provides an overview of the agreement between the predicted dose, optimized dose, and clinical dose across various disease sites, as shown in Figure 4. Further analysis includes DVH metric comparisons for the three most prevalent disease sites, prostate, liver, and brain, with both absolute/relative DVH values summarized in Table 1. The corresponding MAPEs for these sites are provided in Tables S5, S7, and S9.

**TABLE 1:** The statistical comparison of DVH metrics of prediction (Pred), opt‐plan (Opt), and clinical dose (Clinical) of prostate, liver, and brain cases. Bolded values indicate the minimum mean DVH metric values for each OAR.

Prostate cases						
	Pred		Opt		Clinical	
	$D_{95\%}/Rx$ (%)	$D_{5\%}/Rx$ (%)	$D_{95\%}/Rx$ (%)	$D_{5\%}/Rx$ (%)	$D_{95\%}/Rx$ (%)	$D_{5\%}/Rx$ (%)
PTV	103.19 ± 4.84	115.27 ± 12.70	101.72 ± 4.14	119.45 ± 12.57	101.35 ± 2.69	116.64 ± 12.79
	$D_{50\%}$	$D_{2\%}$	$D_{50\%}$	$D_{2\%}$	$D_{50\%}$	$D_{2\%}$
Bladder	7.46 ± 8.02	39.41 ± 7.66	**5.86** ± **6.00**	**37.73** ± **8.17**	6.71 ± 5.62	38.75 ± 6.47
Rectal wall	14.49± 5.96	39.37 ± 6.07	**10.42** ± **4.56**	37.90 ± 6.91	10.83 ± 4.07	**37.73** ± **6.95**
Urethra	42.73 ± 7.66	44.95 ± 6.02	44.43 ± 8.03	46.74 ± 6.68	**40.05** ± **7.17**	**41.78** ± **6.33**
Femoral head	9.72 ± 4.00	16.22 ± 4.17	**7.24** ± **3.40**	**13.24** ± **3.61**	9.92 ± 4.98	17.60 ± 4.81
**Liver cases**						
	**Pred**		**Opt**		**Clinical**	
	$D_{95\%}/Rx$ (%)	$D_{5\%}/Rx$ (%)	$D_{95\%}/Rx$ (%)	$D_{5\%}/Rx$ (%)	$D_{95\%}/Rx$ (%)	$D_{5\%}/Rx$ (%)
PTV	100.55 ± 1.67	115.54 ± 5.11	99.88 ± 0.80	121.43 ± 3.15	100.92 ± 5.09	117.11 ± 12.16
	$D_{50\%}$	$D_{2\%}$	$D_{50\%}$	$D_{2\%}$	$D_{50\%}$	$D_{2\%}$
Bowel	1.89 ± 2.15	10.16 ± 10.69	**1.23** ± **1.92**	**8.70** ± **9.79**	1.98 ± 2.15	10.50 ± 10.41
Kidney	2.62 ± 3.25	8.84 ± 6.64	**1.78** ± **2.65**	**7.26** ± **6.23**	2.56 ± 3.03	9.57 ± 7.39
Stomach	6.03 ± 5.66	18.43 ± 14.86	**4.43** ± **4.65**	15.75 ± 14.90	6.21 ± 5.86	**15.67** ± **11.99**
Esophagus	8.62 ± 6.93	14.62 ± 10.30	**6.77** ± **6.07**	**12.36** ± **9.30**	10.77 ± 8.39	17.19 ± 11.59
**Brain cases**						
	**Pred**		**Opt**		**Clinical**	
	$D_{95\%}/Rx$ (%)	$D_{5\%}/Rx$ (%)	$D_{95\%}/Rx$ (%)	$D_{5\%}/Rx$ (%)	$D_{95\%}/Rx$ (%)	$D_{5\%}/Rx$ (%)
PTV	97.55 ± 19.01	117.35 ± 27.81	97.81 ± 18.92	120.61 ± 28.49	96.43 ± 18.46	115.86 ± 27.10
	$D_{50\%}$	$D_{2\%}$	$D_{50\%}$	$D_{2\%}$	$D_{50\%}$	$D_{2\%}$
Brainstem	6.99 ± 5.83	15.28 ± 9.04	**5.79** ± **4.77**	**13.30** ± **7.88**	7.02 ± 5.36	14.89 ± 8.61
Optic pathway	6.53 ± 6.15	11.75 ± 10.16	**5.85** ± **5.46**	**10.53** ± **9.24**	6.20 ± 5.86	10.83 ± 8.71
Hippocampus	4.68 ± 3.02	7.17 ± 4.30	4.18 ± 2.76	6.70 ± 4.07	**3.86** ± **3.28**	**5.76** ± **4.54**

To assess the quality of the predicted dose, we first analysis the agreement between predicted and optimized dose. As shown in Figure 4, the average GPR across the test dataset was 92.36%, indicating strong overall agreement between the predicted and optimized doses. Although certain disease sites, such as the lung, showed a broader range of GPR values, most sites, including prostate, liver, and pancreas, exhibited consistently high GPR performance. Specifically, for prostate cases (*n* = 46), liver (*n* = 21), and brain (*n* = 18) cases, the MAPEs of (PTV $D_{95\%}$, PTV $D_{5\%}$) were (1.51%, 4.55%), (0.80%, 6.70%), and (1.22%, 4.17%), respectively. Across these three sites, the MAPE of OARs’ $D_{50\%}$ and $D_{5\%}$ values remained within 10% (See details in Tables S5, S7, and S9). When comparing the predicted and optimized doses with the clinical dose, a wider range of GPR values was observed (Figure 4), with an average GPR of 86.13% on the test dataset. In the DVH metric comparison, the PTV‐related metrics showed good agreement with the clinical doses, which was also observed for SIB cases, as summarized in Tables S10 and S11, with MAPE remaining within 10% across predicted, optimized, and clinical plans. The optimized dose achieved a lower $D_{50\%}$ and $D_{2\%}$ values for most OARs across the three major disease sites, as detailed in Table 1.

**FIGURE 5:**
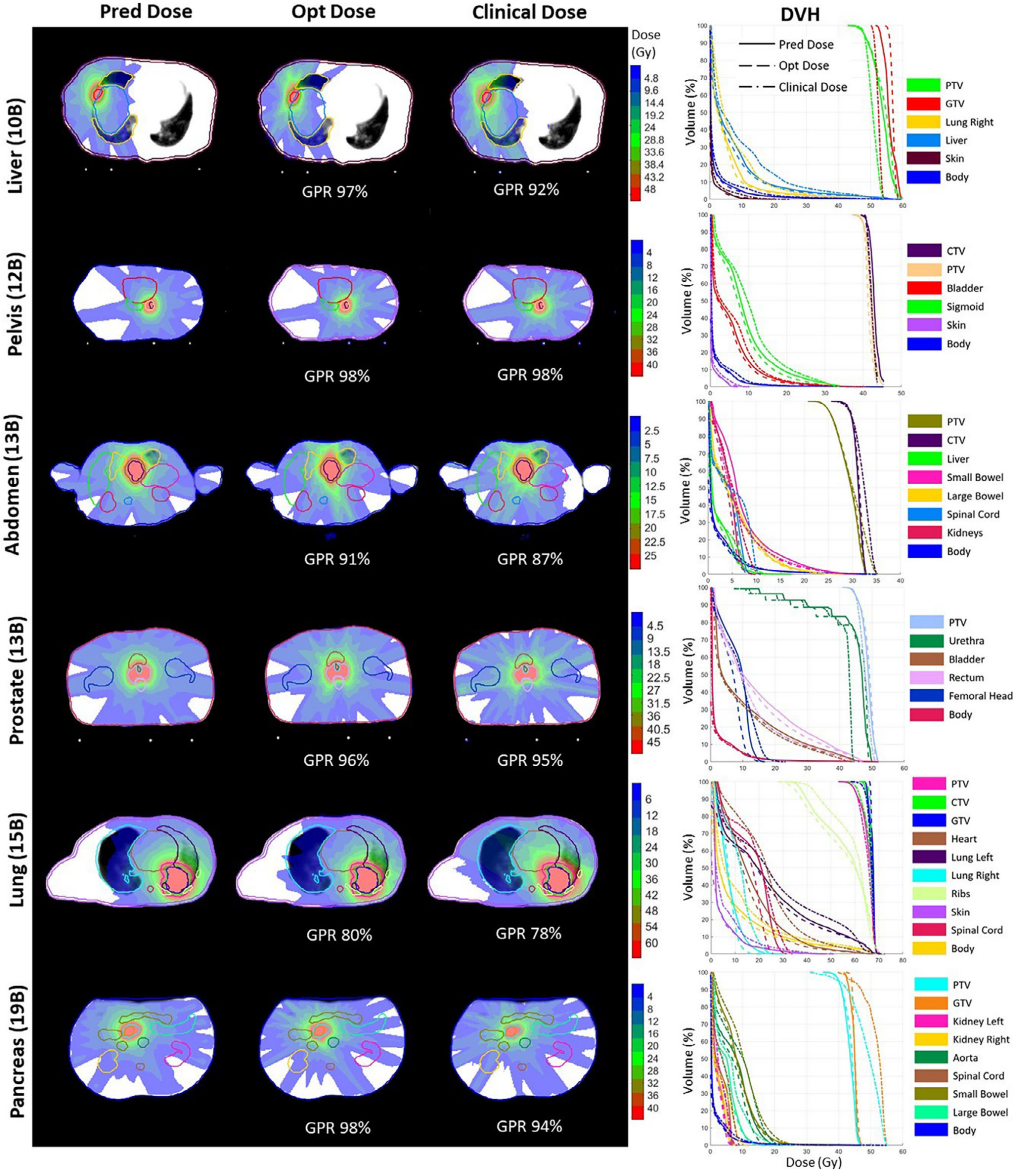
The comparison of dose distributions and DVHs of the predicted, optimized and the clinical dose for six different disease sites with different beam numbers (e.g., 10B denotes 10 beams). The GPR with 3%/2 mm criteria and a 10% LDT were calculated between predicted and optimized or clinical doses.

**FIGURE 6:**
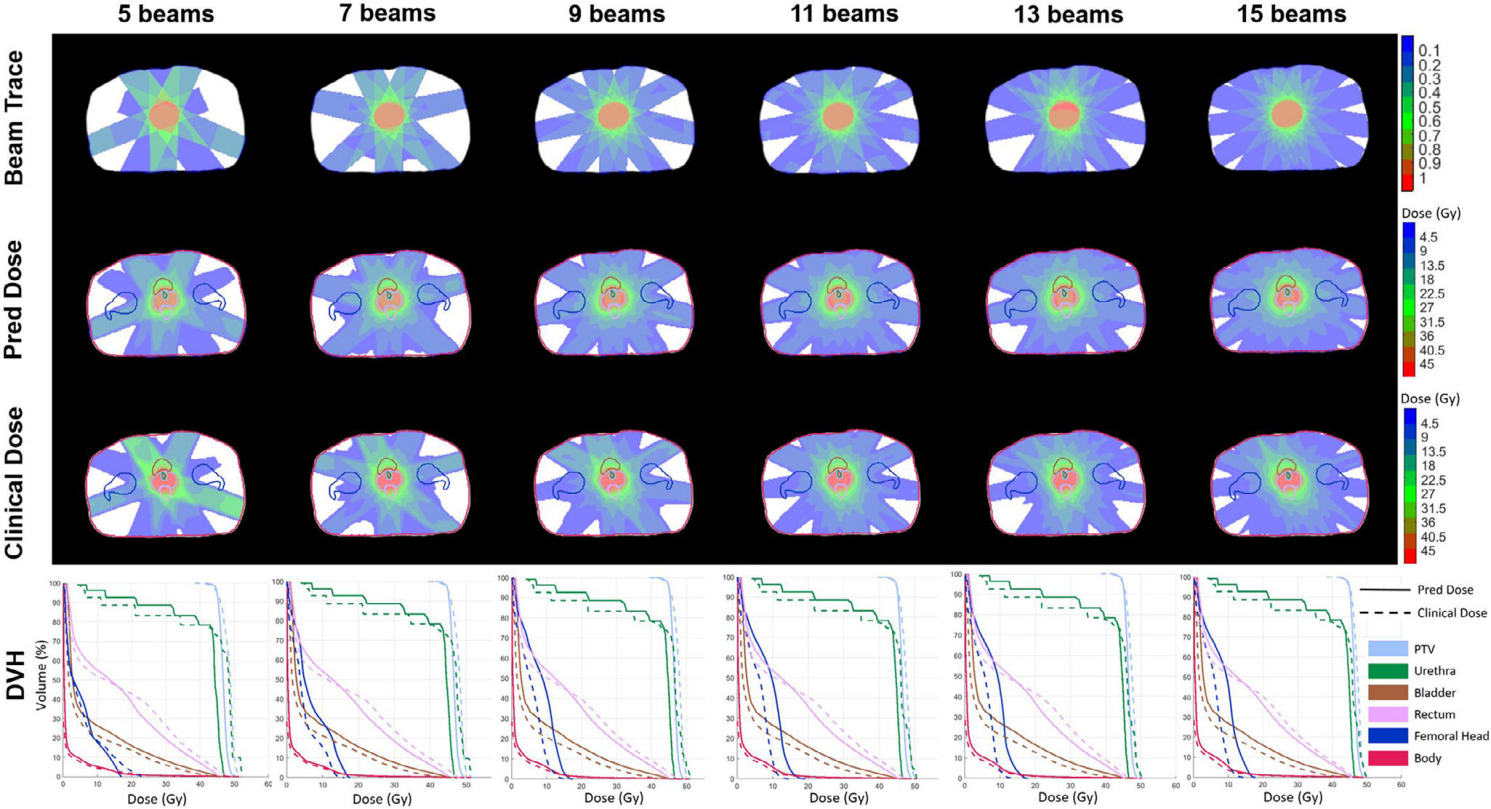
UniDose Predicted dose (Pred Dose) and clinical dose (Clinical Dose) distributions and DVHs for a representative prostate case under six different beam configurations, as illustrated by the beam‐trace inputs (Beam Trace).

Additional case studies across six different disease sites with diverse beam configurations are presented in Figure 5. The predicted and optimized doses exhibited similar dose patterns along the beam paths, and their corresponding DVH curves showed strong consistency. Together, the case studies and statistical results suggest that the predicted dose is generally approachable by dose optimization. While the dose patterns along the beam paths were largely consistent among the predicted, optimized, and clinical doses, slight differences were observed in specific cases. For example, as shown in Figure 5, the lung case, both the predicted and optimized doses achieved improved sparing of the right lung and heart compared to the clinical dose, as also reflected in the DVH curves. Furthermore, DVH comparisons in most cases showed that the predicted and optimized doses achieved a better balance between target coverage and OAR sparing. To further demonstrate the flexibility and generalization capability of UniDose for arbitrary beam configurations, Figure 6 presents a representative prostate case evaluated under six beam configurations ranging from sparse to dense angular sampling. UniDose‐predicted dose distributions were compared with manually generated clinical plans using identical beam configurations. As shown in Figure 6, the predicted doses closely matched the main dose deposition patterns of the clinical plans, with strong agreement in the DVH curves across all configurations. Notably, despite limited or even unseen low‐beam‐number cases in the training dataset, UniDose maintained robust performance under sparse beam arrangements, supporting its ability to generalize beyond beam configurations observed during training.

**FIGURE 7:**
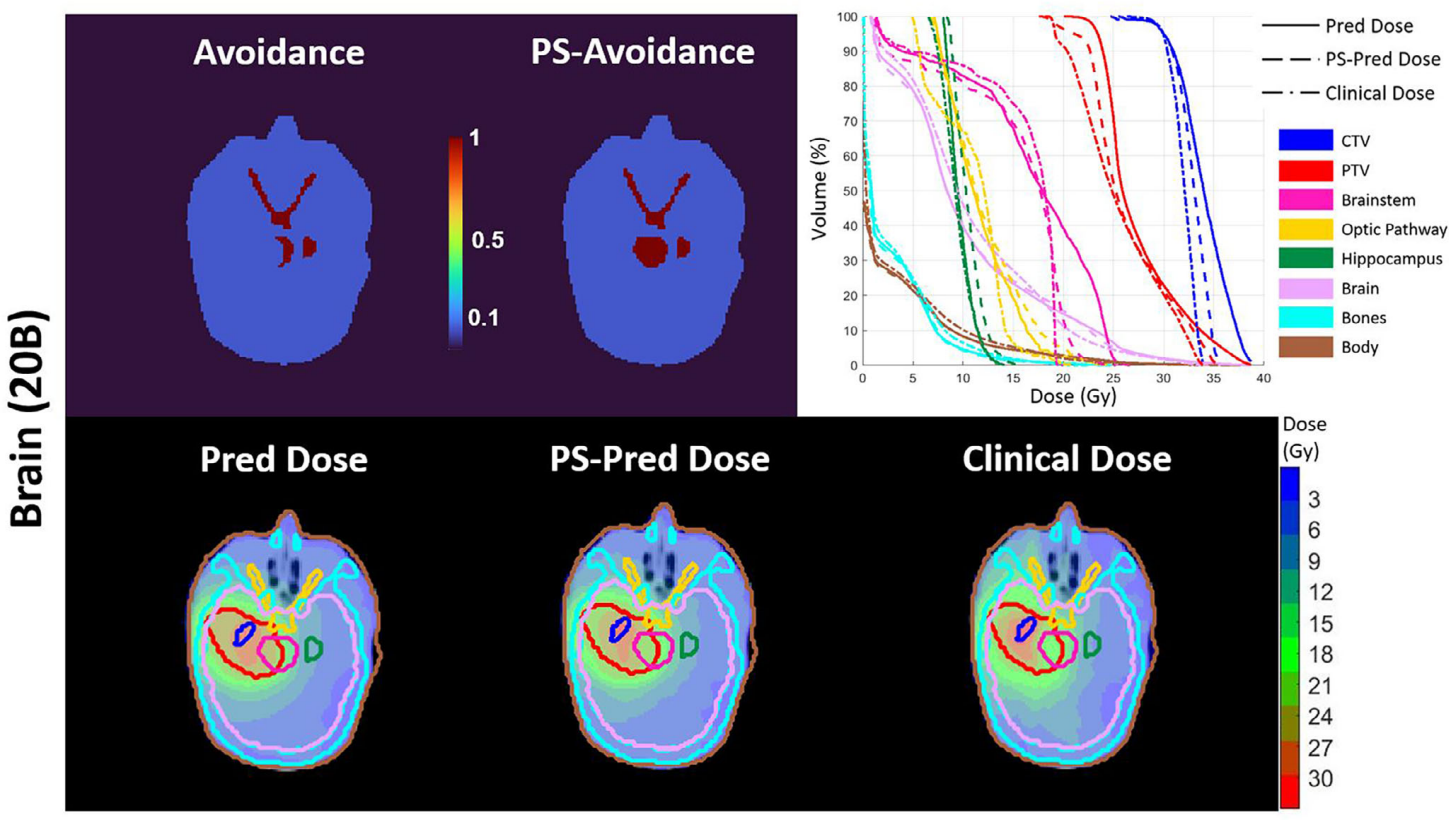
Dose distributions and DVHs with predicted dose (Pred Dose), patient‐specific predicted dose (PS‐Pred Dose) and Clinical Dose for a brain case with 20 beams.

However, the predicted dose distribution can be improved for certain challenge treatment cases with additional inputs. One challenging scenario arises when the tumor encases or is adjacent to critical structures. For instance, in the prostate case where the urethra passes through the tumor, the current predicted dose distribution could be improved by blocking beam paths associated with the urethra to reduce the dose to it. Another challenge is to accommodate patient‐specific dose requirements, which often presents as outliers. Figure 7 illustrates such a scenario in a brain case, where the tumor overlaps with the brainstem. The clinical plan sacrifices part of the tumor to protect the brainstem, whereas the original UniDose prediction prioritized tumor coverage. To better reflect patient‐specific trade‐offs, we modified the avoidance input channel by labeling the overlapping region as OAR instead of the target. With this adjustment, the UniDose prediction aligned more closely with the clinical dose in both spatial distribution and DVH curves.

## 4 DISCUSSION

This study proposes a universal and novel dose prediction framework, UniDose, capable of learning from a highly heterogeneous dataset with respect to both beam configurations and disease sites by incorporating anatomy and beam information into the network. To the best of our knowledge, this is the first study to report a generalizable dose prediction model evaluated on such a diverse dataset, spanning 25 disease sites with a range of beam configurations. This work demonstrates the feasibility of developing a single model applicable across diverse clinical scenarios, representing a meaningful advancement toward practical and scalable automated treatment planning.

The advantages of UniDose arise from two key factors: the automated configuration capabilities of nnU‐Net and the standardized data preparation pipeline. An essential superiority of nnU‐Net lies in its combination of generalizability and ease of use, which distinguishes it from more complex generative adversarial networks (GANs)^12^, ^31^ and diffusion models.^32^, ^33^ Built upon the nnU‐Net architecture, UniDose inherits a core strength of automated configuration. This feature enables the model to dynamically adapt to new datasets by extracting dataset‐specific properties and applying heuristic rules to configure preprocessing, data augmentation, and network parameters without manual intervention. Such automation ensures robust performance across a wide range of disease sites and beam configurations, as demonstrated in this study. In contrast, GAN‐ and diffusion‐based approaches often require significant task‐specific customization. These models typically involve elaborate architecture design, careful hyperparameter tuning, and iterative inference processes that are computationally intensive. While generative models have shown promise in producing high‐fidelity dose distributions, their clinical deployment is limited by implementation complexity, the need for expert tuning, and a lack of inherent adaptability to heterogeneous datasets and variable treatment conditions. Additionally, prior studies have pointed out that proper data preparation and preprocessing are often more critical for unlocking the potential of convolutional neural networks than architectural modifications.^16^, ^25^ In this study, the three input channels, prescription, avoidance, and beam trace, were carefully constructed to encode clinically meaningful information while maintaining generalizability across heterogeneous datasets. The use of a normalized prescription map ensures consistent representation of planning goals, while the unified avoidance mask consolidates multiple OARs into a single weighted input, improving practicality for partially labeled clinical datasets. Regarding the impact of different weight assignments in the avoidance channel, our prior study^7^ reported that applying equal weights during inference increased the MAPE for most OARs. Therefore, we recommend that weight assignment should, first, follow general clinical requirements and, second, remain consistent between the training and inference stages. Moreover, the cumulative beam trace image offers a compact and standardized dose‐domain representation of multi‐beam geometry without requiring complex fluence modeling. This well‐structured preprocessing pipeline reduces variability across cases, which is critical for robust and scalable prediction across diverse treatment scenarios.

Current results demonstrate that UniDose predictions are physically feasible, as evidenced by high consistency in both GPR and DVH comparisons, and clinically meaningful, as the DVH metrics closely match those of the clinical dose distributions. In addition to the overall strong performance, the results indicate that predictions for certain cases exhibit greater variation from the clinical dose or moderately reduced GPR values. For highly heterogeneous sites such as the lung, this variation is primarily attributable to the exclusion of density information (e.g., computed tomography [CT]) from the current input design. In cases with substantial tissue heterogeneity, particularly lung cases with large air cavities, the model has limited ability to account for density‐induced dose perturbations, leading to increased variability in GPR values (Figure 4). Notably, similar limitations were observed in our previous work,^11^ where a U‐Net‐based model trained exclusively on lung IMRT cases with diverse beam configurations, but without CT input, also demonstrated residual discrepancies relative to clinical doses, indicating that site‐specific training alone is insufficient to fully address this challenge. Incorporating CT images as an additional input channel in future work may therefore improve prediction accuracy in highly heterogeneous regions. For brain and abdomen cases, representative low‐GPR examples are provided in Figures S2 and S3 to facilitate further interpretation. In these sites, the reduced GPR values (GPR < 90%) mainly arise from the diversity of clinical scenarios included in the dataset, such as stereotactic body radiotherapy (SBRT) treatments, inconsistency of both target number and location, and patient‐specific planning requirements as exemplified by an out‐of‐distribution case in Figure 7. A limitation of the current UniDose framework is that it does not explicitly encode case‐specific constraints and/or planner preferences. Consequently, reproducing highly individualized modulation patterns present in some clinical plans can be challenging, leading to lower Pred versus Clinic GPR. In addition, for Pred versus Opt comparisons, the smoother dose distributions produced by convolutional neural networks, such as U‐Net‐based architectures that learn population‐averaged dose patterns, may not fully capture the strong local modulation, further contributing to reduced GPR. Importantly, GPR is a stringent voxel‐wise agreement metric originally designed for comparing planned and delivered doses, and moderate reductions in GPR do not directly imply poor predictive performance in the context of dose prediction. Even in lower‐GPR cases shown in Figures S2 and S3, the predicted doses demonstrate good agreement in overall dose deposition patterns and DVH curves. To further investigate the impact of beam number on model performance, the DVH comparisons in Tables S4–S9 also report DVH metrics and MAPE for cases grouped by the number of beams, for example, prostate cases with 11–14 beams and those with 15–20 beams. The results show that the dosimetric accuracy of UniDose predictions remains comparable across different beam configurations, indicating the robustness of UniDose to variations in multi‐beam geometry.

Moreover, it is important to note that the variation between predicted and clinical doses does not necessarily indicate poor model performance. Treatment plans are often Pareto‐optimal solutions, and multiple dose distributions may be acceptable depending on clinical trade‐offs. Whether a predicted dose distribution is acceptable still requires evaluation by the treatment planner. The primary value of a prediction tool like UniDose is its ability to significantly accelerate the planning process by providing a high‐quality starting point, enabling planners to focus on validation and fine‐tuning rather than creating a plan from scratch. To further improve the generality and patient‐specific adaptability of UniDose, incorporating explicit patient‐specific dose constraints into the model represents an important direction for future work, and a recent study^34^ on a user‐preference‐conditioned dose prediction framework provides a promising pathway toward addressing this limitation. Another promising strategy for handling challenging or patient‐specific cases is to integrate dose prediction with iterative auto‐planning frameworks.^35^, ^36^ In such a hybrid workflow, the predicted dose can serve as a high‐quality initialization or hot start for subsequent auto‐planners, which can then iteratively refine the plan to satisfy individualized clinical objectives. This approach leverages the complementary strengths of data‐driven dose prediction, rapid generation of clinically meaningful dose patterns, and iterative planning algorithms, which excel at fine‐tuning trade‐offs through explicit objective adjustment. Rather than replacing algorithm‐based planning, UniDose is well‐suited to function as an efficient front‐end that reduces planning complexity and accelerates convergence toward patient‐specific optimal solutions. Moreover, a promising future application of UniDose is to support beam configuration selection for IMRT, as it is capable of efficiently predicting dose distributions for different beam directions (multi‐beam geometries). By rapidly generating 3D dose predictions for multiple candidate beam arrangements, UniDose could provide valuable guidance to planners in identifying optimal configurations.

Although UniDose is currently designed for dose prediction with discrete beams, a potential future direction is to extend it to arc therapy by representing the dynamic arc delivery as a set of discrete beams within the beam trace input channel. Additionally, it should be noted that the current optimized doses were generated through FMO only. After final dose calculation, including MLC sequencing and machine‐specific delivery constraints, the final plan quality may change slightly due to the additional physical limitations introduced in this step. In future work, we aim to integrate dose prediction with dose calculation under real machine constraints to develop a fully automated, end‐to‐end planning workflow.

## 5 CONCLUSION

In this study, we proposed UniDose, a universal DL–based dose prediction framework designed to generalize across diverse disease sites and beam configurations. Built upon a customized nnU‐Net architecture and trained with generalized input representations, UniDose demonstrated high prediction accuracy, strong agreement with optimized and clinical doses, as evaluated on a large and heterogeneous dataset. By enabling efficient and accurate dose estimation, UniDose has the potential to serve as a practical tool for real‐world radiotherapy planning and to support further automation in treatment personalization.

## CONFLICT OF INTEREST STATEMENT

The authors have no relevant conflicts of interest to disclose.

## Supporting information



Supporting information

Supporting information

Supporting information

Supporting information
