# Supplementary material for: A universal framework for IMRT dose prediction
Source: Med Phys. 2026 Mar 15;53(3):e70384. doi: 10.1002/mp.70384 (PMC12989320; doi:10.1002/mp.70384)
Supplement: Supplementary file 1 — Supporting information [file MP-53-0-s003.pdf]

Supplementary Material

This document contains supplementary results to accompany the manuscript named: “A Universal Framework for IMRT Dose Prediction”.

- **Detailed Dataset Composition for Training, Validation and Testing Cohorts**

Table S1. Detailed dataset composition for the training cohort, including the number of cases per disease site, the number of simultaneous integrated boost (SIB) and non-SIB plans, PTV boost volume and PTV volume (mean ± SD), and beam number (mean ± SD).

Disease sites	Case Num.	SIB		PTV Boost Volume	PTV Volume	Beam Num.
		True	False			
Prostate	183	122	61	36.6 (40.0)	68.6 (61.6)	14 (2.8)
Liver	99	4	95	31.7 (50.9)	40.1 (83.7)	13 (1.9)
Brain	66	32	34	28.7 (47.2)	105.7 (99.4)	12 (2.4)
Pelvis	42	21	21	23.8 (39.3)	54.7 (73.9)	14 (2.7)
Pancreas	39	27	12	29.2 (52.1)	98.1 (87.3)	13 (2.0)
Abdomen	31	9	22	37.5 (48.5)	213.7 (212.9)	13 (2.8)
Lung	22	0	22	-	110.0 (159.2)	14 (2.8)
Kidney	21	2	19	27.1 (9.7)	103.1 (76.2)	13 (2.6)
Others	83	22	61	53.8 (75.5)	95.9 (159.6)	13 (3.7)

Table S2. Detailed dataset composition for the validation cohort, including the number of cases per disease site, the number of simultaneous integrated boost (SIB) and non-SIB plans, PTV boost volume and PTV volume (mean ± SD), and beam number (mean ± SD).

Disease sites	Case Num.	SIB		PTV Boost Volume	PTV Volume	Beam Num.
		True	False			
Prostate	43	32	11	42.3 (35.2)	75.9 (25.7)	14 (2.9)
Liver	18	1	17	30.1 (58.5)	55.3 (24.3)	13 (2.1)
Brain	24	12	12	37.2 (58.7)	102.8 (104.9)	11 (1.7)
Pelvis	11	8	3	15.7 (33.1)	75.5 (42.7)	14 (2.0)
Lung	11	0	11	-	101.2 (186.8)	17 (4.0)
Pancreas	12	9	3	37.6 (62.6)	168.2 (84.6)	13 (2.1)
Abdomen	9	3	6	2.6 (1.1)	34.2 (85.0)	14 (1.7)
Others	19	8	11	44.7 (95.5)	163.6 (159.4)	14 (2.3)

Table S3. Detailed dataset composition for the testing cohort, including the number of cases per disease site, the number of simultaneous integrated boost (SIB) and non-SIB plans, PTV boost volume and PTV volume (mean ± SD), and beam number (mean ± SD).

Disease sites	Case Num.	SIB		PTV Boost Volume	PTV Volume	Beam Num.
		True	False			
Prostate	46	28	18	43.2 (34.2)	67.5 (54.5)	14 (2.8)
Liver	21	3	18	33.7 (22.5)	45.2 (55.7)	13 (2.2)
Brain	18	6	12	27.5 (73.4)	99.3 (65.2)	11 (2.8)
Pelvis	12	6	6	18.2 (29.6)	44.5 (17.1)	14 (3.1)
Lung	9	0	9	-	112.7 (233.2)	16 (1.1)
Pancreas	7	3	4	10.3 (42.1)	35.3 (48.8)	13 (2.3)
Abdomen	5	2	3	32.4 (12.9)	255.5 (129.0)	13 (1.3)
Others	20	5	15	52.5 (89.9)	129.9 (528.3)	14 (2.2)

- **DVH metrics with MAPE for Prostate Cases**

Table S4. The statistical comparison of DVH metrics of predicted (Pred), optimized (Opt), and clinical dose (Clin) of prostate cases with 11 to 14 beams (32 cases), 15 to 20 beams (14 cases) and total 46 cases (All).

DVH metrics (mean ± std.)									
	Beam 11-14			Beam 15-20			All		
	Pred	Opt	Clin	Pred	Opt	Clin	Pred	Opt	Clin
PTV									
$D_{95\%}/Rx$ (%)	103.45 ± 5.06	101.81 ± 4.44	101.41 ± 2.98	102.53 ± 4.28	101.49 ± 3.33	101.20 ± 1.80	103.19 ± 4.84	101.72 ± 4.14	101.35 ± 2.69
$D_{5\%}/Rx$ (%)	113.39 ± 9.00	117.20 ± 9.64	115.27 ± 9.65	120.09 ± 18.63	125.21 ± 16.99	120.15 ± 18.44	115.27 ± 12.70	119.45 ± 12.57	116.64 ± 12.79
Bladder									
$D_{50\%}$ (Gy)	4.29 ± 5.31	**3.92 ± 4.76**	6.38 ± 7.67	11.38 ± 8.10	**8.00 ± 4.89**	10.29 ± 5.01	6.99 ± 7.24	**5.47 ± 5.11**	6.99 ± 7.24
$D_{2\%}$ (Gy)	34.51 ± 10.14	**31.93 ± 9.57**	35.49 ± 9.52	37.56 ± 3.96	**35.50 ± 4.12**	38.43 ± 2.44	35.67 ± 8.34	**33.29 ± 8.00**	36.61 ± 7.65
Bladder Wall									
$D_{50\%}$ (Gy)	5.48 ± 6.41	**4.50 ± 5.89**	4.68 ± 3.89	13.22 ± 10.66	9.93 ± 7.80	**9.46 ± 5.05**	7.72 ± 8.50	6.07 ± 6.49	**6.06 ± 4.73**
$D_{2\%}$ (Gy)	40.21 ± 5.89	**38.66 ± 6.53**	38.93 ± 5.08	44.57 ± 7.14	43.92 ± 7.92	**42.38 ± 5.90**	41.47 ± 6.49	40.18 ± 7.26	**39.93 ± 5.48**
Rectal Wall									
$D_{50\%}$ (Gy)	12.95 ± 4.91	**9.11 ± 3.50**	9.94 ± 3.93	17.96 ± 6.83	13.37 ± 5.42	**12.83 ± 3.79**	14.49 ± 5.96	**10.42 ± 4.56**	10.83 ± 4.07
$D_{2\%}$ (Gy)	38.14 ± 5.62	36.27 ± 6.06	**35.84 ± 6.48**	42.12 ± 6.37	**41.56 ± 7.56**	41.96 ± 6.28	39.37 ± 6.07	37.90 ± 6.91	**37.73 ± 6.95**
Urethra									
$D_{50\%}$ (Gy)	42.27 ± 8.84	43.77 ± 9.24	**39.52 ± 8.24**	43.94 ± 2.82	46.19 ± 2.82	**41.43 ± 2.64**	42.73 ± 7.66	44.43 ± 8.03	**40.05 ± 7.17**
$D_{2\%}$ (Gy)	44.41 ± 6.72	45.89 ± 7.45	**41.35 ± 7.24**	46.36 ± 3.48	48.99 ± 3.27	**42.93 ± 2.77**	44.95 ± 6.02	46.74 ± 6.68	**41.78 ± 6.33**
Femoral Head									
$D_{50\%}$ (Gy)	8.40 ± 2.84	**5.99 ± 2.51**	7.99 ± 2.77	12.94 ± 4.65	**10.29 ± 3.44**	14.63 ± 6.04	9.72 ± 4.00	**7.24 ± 3.40**	9.92 ± 4.98
$D_{2\%}$ (Gy)	15.00 ± 2.94	**12.04 ± 2.63**	16.04 ± 2.65	19.17 ± 5.27	**16.16 ± 4.09**	21.40 ± 6.63	16.22 ± 4.17	**13.24 ± 3.61**	17.60 ± 4.81

* Bolded values indicate the minimum mean DVH metric values for each organ-at-risk (OAR).

Table S5. Mean absolute percent error (MAPE) for the relevant DVH metrics for predicted and clinical dose (Pred-Clin), and predicted and optimized doses (Pred-Opt) of prostate cases with 11 to 14 beams (32 cases), 15 to 20 beams (14 cases) and total 46 cases (All).

Mean absolute percent error (MAPE) for DVH metrics						
	Beam 11-14		Beam 15-20		All	
	Pred-Clin	Pred-Opt	Pred-Clin	Pred-Opt	Pred-Clin	Pred-Opt
PTV						
$D_{95\%}$	2.04%	1.65%	2.10%	1.15%	2.06%	1.51%
$D_{5\%}$	3.47%	4.25%	3.32%	5.30%	3.43%	4.55%
Bladder						
$D_{50\%}$	5.03%	1.09%	8.32%	7.50%	6.28%	3.55%
$D_{2\%}$	4.28%	6.12%	4.91%	4.08%	4.52%	5.62%
Bladder Wall						
$D_{50\%}$	4.66%	2.50%	11.52%	7.46%	6.65%	3.93%
$D_{2\%}$	4.29%	4.26%	5.63%	1.78%	4.68%	4.05%
Rectal Wall						
$D_{50\%}$	9.18%	9.30%	12.88%	10.31%	10.32%	9.61%
$D_{2\%}$	6.44%	5.48%	1.52%	3.19%	4.93%	4.77%
Urethra						
$D_{50\%}$	6.55%	4.47%	5.77%	5.30%	6.34%	4.70%
$D_{2\%}$	7.66%	4.30%	7.74%	6.20%	7.68%	4.83%
Femoral Head						
$D_{50\%}$	1.76%	5.69%	4.74%	5.84%	2.63%	5.74%
$D_{2\%}$	3.31%	7.01%	7.40%	6.63%	4.50%	6.90%

- **DVH metrics with MAPE for Liver Cases**

Table S6. The statistical comparison of DVH metrics of predicted (Pred), optimized (Opt), and clinical dose (Clin) of liver cases with 10 to 13 beams (15 cases), 14 to 18 beams (6 cases) and total 21 cases (All).

DVH metrics (mean ± std.)									
	Beam 10-13			Beam 14-18			All		
	Pred	Opt	Clin	Pred	Opt	Clin	Pred	Opt	Clin
PTV									
$D_{95\%}/Rx$ (%)	100.58 ± 1.82	100.07 ± 0.64	99.72 ± 1.01	100.50 ± 1.43	99.49 ± 0.99	103.31 ± 8.56	100.55 ± 1.67	99.88 ± 0.80	100.92 ± 5.09
$D_{5\%}/Rx$ (%)	115.78 ± 5.51	121.35 ± 2.76	115.53 ± 11.58	115.07 ± 4.51	121.58 ± 4.04	120.28 ± 13.46	115.54 ± 5.11	121.43 ± 3.15	117.11 ± 12.16
Bowel									
$D_{50\%}$ (Gy)	1.39 ± 1.78	**0.71 ± 1.22**	1.40 ± 1.72	2.65 ± 2.51	**2.01 ± 2.51**	2.87 ± 2.50	1.89 ± 2.15	**1.23 ± 1.92**	1.98 ± 2.15
$D_{2\%}$ (Gy)	8.06 ± 9.97	**6.63 ± 8.76**	8.45 ± 10.25	13.32 ± 11.38	**11.81 ± 10.80**	13.57 ± 10.29	10.16 ± 10.69	**8.70 ± 9.79**	10.50 ± 10.41
Kidney									
$D_{50\%}$ (Gy)	1.38 ± 1.58	**0.68 ± 1.29**	1.49 ± 1.76	5.11 ± 4.34	**3.98 ± 3.35**	4.70 ± 3.97	2.62 ± 3.25	**1.78 ± 2.65**	2.56 ± 3.03
$D_{2\%}$ (Gy)	6.05 ± 4.91	**4.37 ± 4.17**	6.23 ± 5.00	14.41 ± 6.32	**13.03 ± 5.78**	16.26 ± 7.00	8.84 ± 6.64	**7.26 ± 6.23**	9.57 ± 7.39
Stomach									
$D_{50\%}$ (Gy)	3.61 ± 3.81	**2.34 ± 2.92**	4.06 ± 4.99	9.96 ± 6.16	**7.84 ± 5.08**	9.70 ± 5.74	6.03 ± 5.66	**4.43 ± 4.65**	6.21 ± 5.86
$D_{2\%}$ (Gy)	13.37 ± 14.20	**11.02 ± 14.84**	12.37 ± 12.85	26.66 ± 12.65	23.45 ± 12.14	**21.04 ± 8.63**	18.43 ± 14.86	15.75 ± 14.90	**15.67 ± 11.99**
Esophagus									
$D_{50\%}$ (Gy)	6.78 ± 5.55	**5.24 ± 4.88**	8.73 ± 6.61	12.01 ± 8.41	**9.57 ± 7.47**	14.51 ± 10.57	8.62 ± 6.93	**6.77 ± 6.07**	10.77 ± 8.39
$D_{2\%}$ (Gy)	11.58 ± 8.57	**9.72 ± 7.81**	14.05 ± 10.09	20.18 ± 11.63	**17.19 ± 10.56**	22.94 ± 12.83	14.62 ± 10.30	**12.36 ± 9.30**	17.19 ± 11.59

* Bolded values indicate the minimum mean DVH metric values for each organ-at-risk (OAR).

Table S7. Mean absolute percent error (MAPE) for the relevant DVH metrics of predicted and clinical plan (Pred-Clin), and predicted and optimized doses (Pred-Opt) of liver cases with 10 to 13 beams (15 cases), 14 to 18 beams (6 cases) and total 21 cases (All).

Mean absolute percent error (MAPE) for DVH metrics						
	Beam 10-13		Beam 14-18		All	
	Pred-Clin	Pred-Opt	Pred-Clin	Pred-Opt	Pred-Clin	Pred-Opt
PTV						
$D_{95\%}$	0.86%	0.51%	4.16%	1.38%	1.96%	0.80%
$D_{5\%}$	8.38%	6.80%	11.60%	6.51%	9.45%	6.70%
Bowel						
$D_{50\%}$	0.58%	1.50%	0.78%	1.35%	0.66%	1.44%
$D_{2\%}$	1.69%	3.29%	5.13%	3.60%	3.07%	3.42%
Kidney						
$D_{50\%}$	0.57%	1.57%	1.90%	2.38%	1.01%	1.84%
$D_{2\%}$	3.07%	3.88%	4.46%	4.02%	3.53%	3.93%
Stomach						
$D_{50\%}$	2.26%	3.07%	1.27%	4.50%	1.88%	3.61%
$D_{2\%}$	4.13%	5.82%	11.43%	6.82%	6.91%	6.20%
Esophagus						
$D_{50\%}$	4.62%	3.61%	5.84%	5.13%	5.05%	4.15%
$D_{2\%}$	5.91%	5.30%	9.67%	6.25%	7.24%	5.64%

- **DVH metrics with MAPE for Brain Cases**

Table S8. The statistical comparison of DVH metrics of prediction (Pred), opt-plan (Opt), and clinical dose (Clin) of brain cases with 9 to 12 beams (13 cases), 13 to 20 beams (5 cases) and total 18 cases (All).

DVH metrics (mean ± std.)									
	Beam 9-12			Beam 13-20			All		
	Pred	Opt	Clin	Pred	Opt	Clin	Pred	Opt	Clin
PTV									
$D_{95\%}/Rx$ (%)	101.11 $\pm$ 3.84	101.14 $\pm$ 2.18	100.32 $\pm$ 0.99	86.85 $\pm$ 37.81	87.81 $\pm$ 38.34	84.74 $\pm$ 36.69	97.55 $\pm$ 19.01	97.81 $\pm$ 18.92	96.43 $\pm$ 18.46
$D_{5\%}/Rx$ (%)	117.73 $\pm$ 10.04	120.32 $\pm$ 9.75	117.23 $\pm$ 10.11	116.22 $\pm$ 56.69	121.46 $\pm$ 58.39	111.75 $\pm$ 54.81	117.35 $\pm$ 27.81	120.61 $\pm$ 28.49	115.86 $\pm$ 27.10
Brainstem									
$D_{50\%}$ (Gy)	5.66 $\pm$ 3.93	**4.75 $\pm$ 3.32**	6.17 $\pm$ 4.48	10.62 $\pm$ 9.11	**8.63 $\pm$ 7.41**	9.34 $\pm$ 7.59	6.99 $\pm$ 5.83	**5.79 $\pm$ 4.77**	7.02 $\pm$ 5.36
$D_{2\%}$ (Gy)	14.86 $\pm$ 8.57	**12.21 $\pm$ 6.84**	14.78 $\pm$ 8.57	16.43 $\pm$ 11.59	16.30 $\pm$ 10.85	**15.18 $\pm$ 10.08**	15.28 $\pm$ 9.04	**13.30 $\pm$ 7.88**	14.89 $\pm$ 8.61
Optic Pathway									
$D_{50\%}$ (Gy)	6.91 $\pm$ 6.07	**6.10 $\pm$ 5.72**	6.40 $\pm$ 6.07	4.87 $\pm$ 5.57	**4.78 $\pm$ 5.03**	5.34 $\pm$ 5.88	6.53 $\pm$ 6.15	**5.85 $\pm$ 5.46**	6.20 $\pm$ 5.86
$D_{2\%}$ (Gy)	12.05 $\pm$ 9.16	**10.61 $\pm$ 9.63**	11.07 $\pm$ 9.16	10.47 $\pm$ 9.83	10.21 $\pm$ 9.13	**9.78 $\pm$ 7.98**	11.75 $\pm$ 10.16	**10.53 $\pm$ 9.24**	10.83 $\pm$ 8.71
Hippocampus									
$D_{50\%}$ (Gy)	3.78 $\pm$ 3.03	3.44 $\pm$ 3.20	**3.11 $\pm$ 3.09**	5.40 $\pm$ 3.14	4.77 $\pm$ 2.56	**4.46 $\pm$ 3.65**	4.68 $\pm$ 3.02	4.18 $\pm$ 2.76	**3.86 $\pm$ 3.28**
$D_{2\%}$ (Gy)	5.46 $\pm$ 4.71	5.31 $\pm$ 4.81	**4.62 $\pm$ 4.87**	8.54 $\pm$ 3.89	7.81 $\pm$ 3.51	**6.67 $\pm$ 4.59**	7.17 $\pm$ 4.30	6.70 $\pm$ 4.07	**5.76 $\pm$ 4.54**

* Bolded values indicate the minimum mean DVH metric values for each organ-at-risk (OAR).

Table S9. Mean absolute percent error (MAPE) of the relevant DVH metrics for predicted and clinical dose (Pred-Clin), and predicted and optimized-dose (Pred-Opt) of brain cases with 9 to 12 beams (13 cases), 13 to 20 beams (5 cases) and total 18 cases (All).

Mean absolute percent error (MAPE) for DVH metrics						
	Beam 9-12		Beam 13-20		All	
	Pred-Clin	Pred-Opt	Pred-Clin	Pred-Opt	Pred-Clin	Pred-Opt
PTV						
$D_{95\%}$	1.92%	1.23%	2.39%	1.17%	2.04%	1.22%
$D_{5\%}$	3.69%	3.82%	4.55%	5.24%	3.90%	4.17%
Brainstem						
$D_{50\%}$	2.62%	3.58%	7.39%	9.58%	3.89%	5.18%
$D_{2\%}$	4.46%	10.40%	6.64%	4.99%	5.04%	8.96%
Optic Pathway						
$D_{50\%}$	2.21%	2.88%	1.58%	1.36%	2.09%	2.60%
$D_{2\%}$	6.34%	5.04%	6.56%	2.92%	6.38%	4.64%
Hippocampus						
$D_{50\%}$	3.18%	2.20%	18.03%	8.34%	11.43%	5.61%
$D_{2\%}$	4.60%	1.33%	18.36%	6.78%	12.25%	4.36%

- **Case Study: A Representative Prostate Case**

Figure S1 illustrates a representative prostate case comparing the UniDose predicted dose, optimized dose, and clinical dose. Although high overall agreement is observed, as reflected by gamma passing rates (GPR) of approximately 90%, the rectal wall $D_{50\%}$ exhibits relatively larger percentage (MAPE) differences between the predicted, optimized, and clinical doses. This behavior arises from two factors. First, the rectal wall is a thin anatomical structure occupying a very limited volume with relatively few voxels. Consequently, moderate absolute dose differences in a small number of voxels can translate into larger percentage errors in $D_{50\%}$ while having minimal impact on global voxel-wise agreement metrics such as GPR. Second, the rectal wall is located adjacent to the posterior boundary of the target and lies within a steep dose-gradient region. In such high-gradient regions, small dose differences in dose distribution can disproportionately affect rectal wall $D_{50\%}$ values.

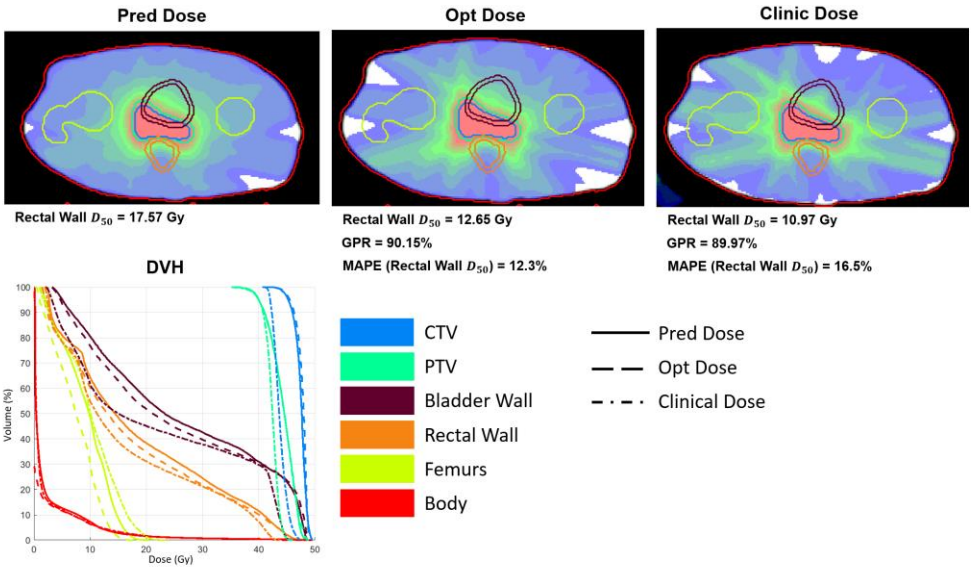

Figure S1. Representative prostate case comparing UniDose-predicted dose (Pred Dose), optimized dose (Opt Dose), and clinical dose (Clinic Dose). Gamma passing rate (GPR) and mean absolute percentage error (MAPE) were evaluated between the Pred Dose and the Opt Dose/Clinic Dose.

- **DVH metrics with MAPE for simultaneous integrated boost (SIB) cases**

In the SIB cohort, prescription dose levels varied across patients in both absolute dose values and the number of prescription levels per plan. To enable consistent evaluation across this heterogeneous dataset, DVH metrics were analyzed in a relative (normalized) form.

For each SIB case, the target with the maximum prescription dose was defined as the boosted targets (PTV boost), while all lower-dose targets were categorized as PTV. The $D_{95\%}$ metric, which reflects target coverage at the intended prescription level, was normalized by the corresponding prescription dose of each target. In contrast, the $D_{5\%}$ metric was normalized by the maximum prescription dose within each case, to account for differences in boost dose levels.

Table S10. The statistical comparison of DVH metrics for the target (PTV) and boosted target (PTV boost) across all SIB cases in the test dataset. Predicted dose (Pred), optimized dose (Opt), and clinical dose (Clinic). Rx^* represents the maximum prescription dose for each case.

	Pred		Opt		Clinic	
	$D_{95\%}/Rx$ (%)	$D_{5\%}/Rx^*$ (%)	$D_{95\%}/Rx$ (%)	$D_{5\%}/Rx^*$ (%)	$D_{95\%}/Rx$ (%)	$D_{5\%}/Rx^*$ (%)
PTV	106.53 ± 6.78	109.86 ± 8.29	108.70 ± 7.10	112.34 ± 8.35	101.45 ± 4.87	105.1 ± 8.39
PTV boost	100.40 ± 3.99	116.13 ± 6.66	102.84 ± 4.14	118.74 ± 7.45	98.38 ± 5.42	109.73 ± 6.13

Table S11. Mean absolute percent error (MAPE) for the relevant DVH metrics for the target (PTV) and boosted target (PTV boost) between predicted dose and optimized dose (Pred-Opt), predicted dose and clinical dose (Pred-Clinic), and optimized dose and clinical dose (Opt-Clinic) for the target volume (PTV) and boost target volume (PTV boost) across all SIB cases in the test dataset. Rx^* represents the maximum prescription dose for each case.

	Pred-Opt		Pred-Clinic		Opt-Clinic	
	$D_{95\%}/Rx$ (%)	$D_{5\%}/Rx^*$ (%)	$D_{95\%}/Rx$ (%)	$D_{5\%}/Rx^*$ (%)	$D_{95\%}/Rx$ (%)	$D_{5\%}/Rx^*$ (%)
PTV	2.16	2.48	6.13	5.69	7.56	7.84
PTV boost	2.54	2.63	3.11	7.14	4.91	9.32

- **Case Study: Brain and Abdomen cases with GPR below 90%**

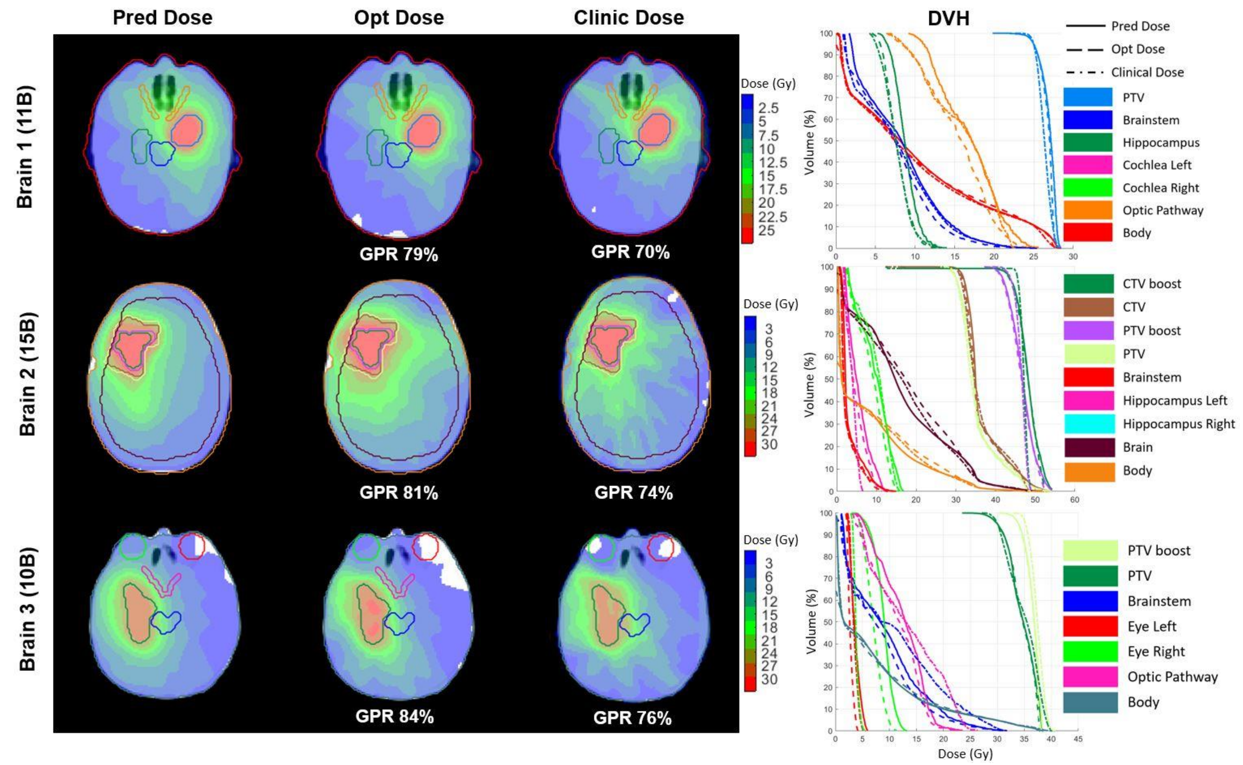

Figure S2. The comparison of dose distributions and DVHs of the predicted (Pred), optimized (Opt) and the clinical (Clinic) dose for three brain cases with different beam numbers (e.g., 10B denotes 10 beams). The GPR with 3%/2mm criteria and a 10% LDT were calculated between predicted and optimized or clinical doses.

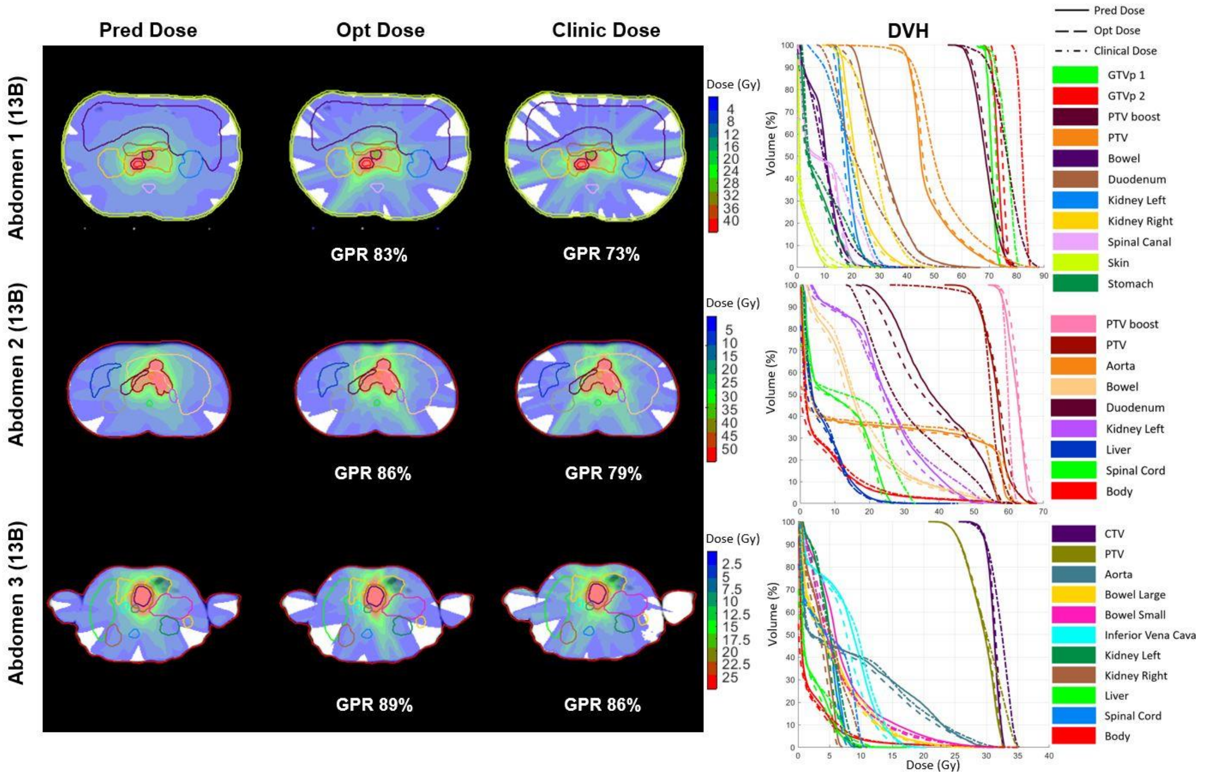

Figure S3. The comparison of dose distributions and DVHs of the predicted (Pred), optimized (Opt) and the clinical (Clinic) dose for three abdomen cases with same beam numbers (e.g., 13B denotes 13 beams). The GPR with 3%/2mm criteria and a 10% LDT were calculated between predicted and optimized or clinical doses.
