# Supplementary figures and images for: A universal framework for IMRT dose prediction
Source: Med Phys. 2026 Mar 15;53(3):e70384. doi: 10.1002/mp.70384 (PMC12989320; doi:10.1002/mp.70384)

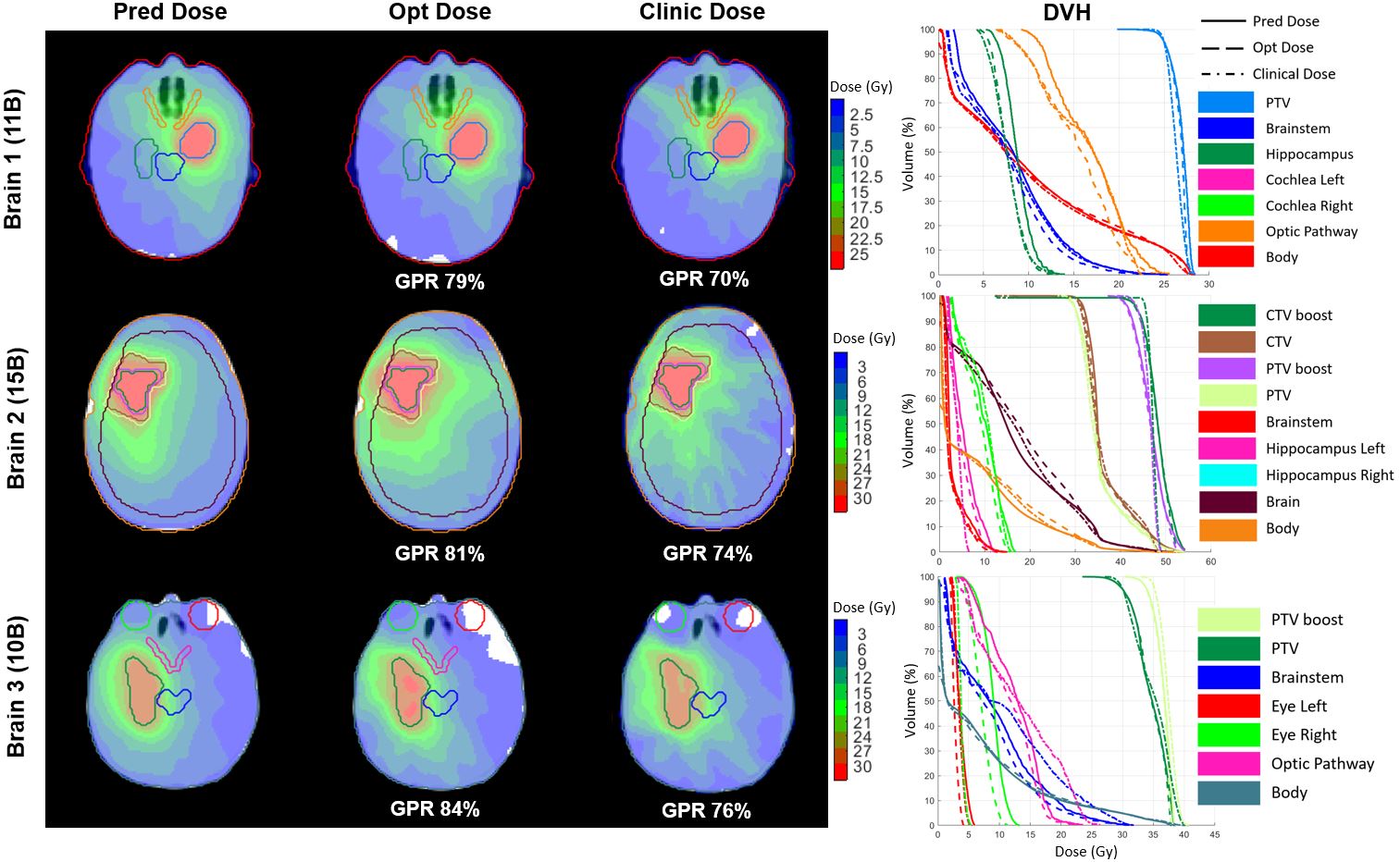

Supplement: Supplementary file 2 — Supporting information [file MP-53-0-s001.JPG]

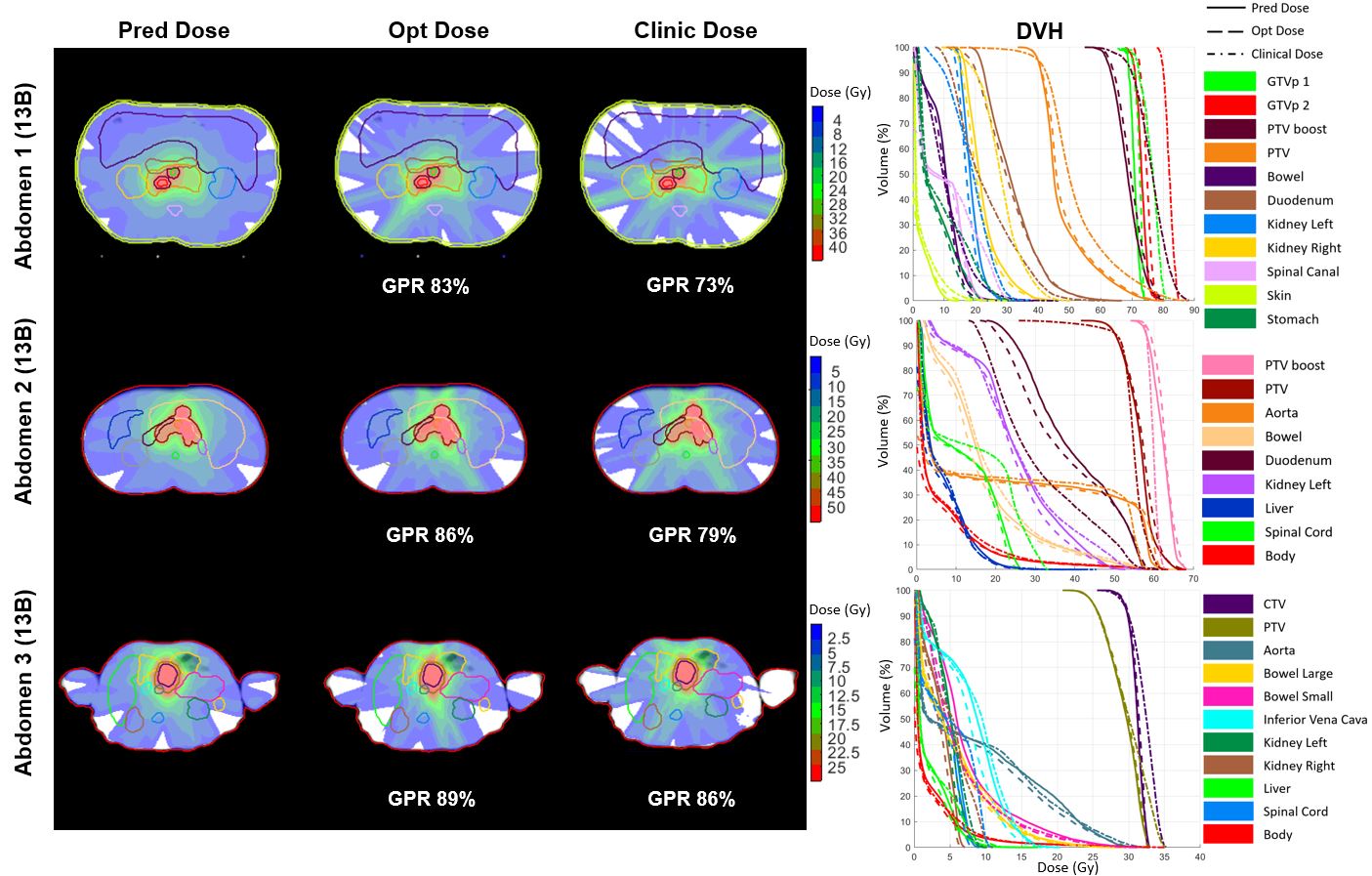

Supplement: Supplementary file 3 — Supporting information [file MP-53-0-s002.JPG]

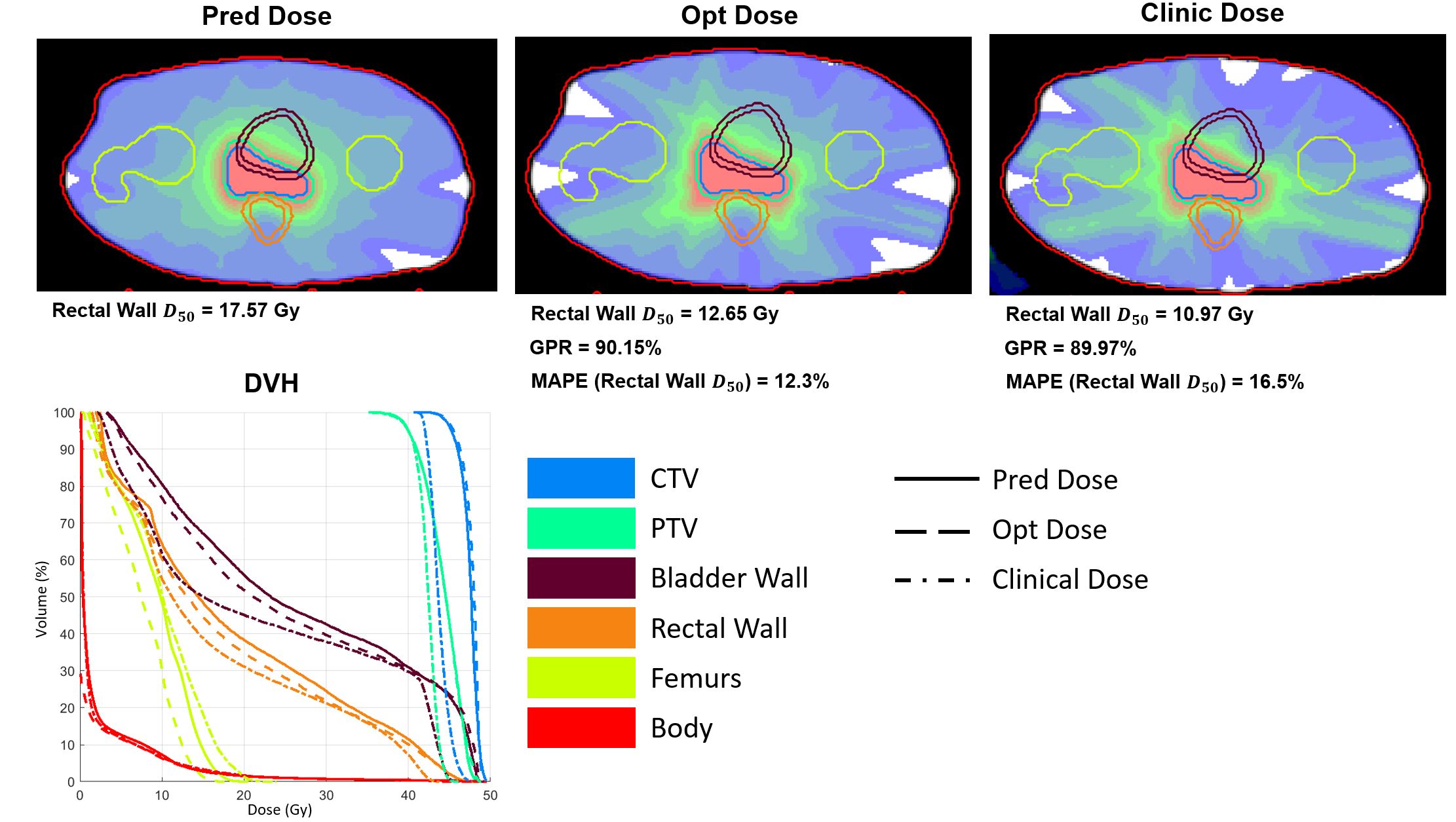

Supplement: Supplementary file 4 — Supporting information [file MP-53-0-s004.JPG]
